# Transfer of Cellular Content from the Allogeneic Cell-Based Cancer Vaccine DCP-001 to Host Dendritic Cells Hinges on Phosphatidylserine and Is Enhanced by CD47 Blockade

**DOI:** 10.3390/cells10113233

**Published:** 2021-11-19

**Authors:** Haoxiao Zuo, Marie-José C. van Lierop, Jorn Kaspers, Remco Bos, Anneke Reurs, Saheli Sarkar, Tania Konry, Alwin Kamermans, Gijs Kooij, Helga E. de Vries, Tanja D. de Gruijl, Alex Karlsson-Parra, Erik H. Manting, Ada M. Kruisbeek, Satwinder Kaur Singh

**Affiliations:** 1 Immunicum, Galileiweg 8, 2333 BD Leiden, The Netherlands; haoxiao.zuo@immunicum.com (H.Z.); jorn.kaspers@immunicum.com (J.K.); remco.bos@immunicum.com (R.B.); anneke.reurs@immunicum.com (A.R.); alex.karlsson-parra@immunicum.com (A.K.-P.); erik.manting@immunicum.com (E.H.M.); ada.kruisbeek@planet.nl (A.M.K.); satwinder.singh@immunicum.com (S.K.S.); 2 Department of Pharmaceutical Sciences, Northeastern University, Boston, MA 02115, USA; sahelisarkar79@gmail.com (S.S.); t.konry@northeastern.edu (T.K.); 3 Department of Molecular Cell Biology and Immunology, Amsterdam University Medical Center, De Boelelaan 1117, 1081HV Amsterdam, The Netherlands; a.kamermans@amsterdamumc.nl (A.K.); g.kooij@amsterdamumc.nl (G.K.); h.devries@amsterdamumc.nl (H.E.d.V.); 4 Department of Medical Oncology, Amsterdam University Medical Center, De Boelelaan 1117, 1081HV Amsterdam, The Netherlands; TD.deGruijl@vumc.nl

**Keywords:** DCP-001, DCOne, allogeneic, dendritic cell, cell-based vaccine, acute myeloid leukemia, intradermal, antigen transfer, CD47, phosphatidylserine

## Abstract

DCP-001 is a cell-based cancer vaccine generated by differentiation and maturation of cells from the human DCOne myeloid leukemic cell line. This results in a vaccine comprising a broad array of endogenous tumor antigens combined with a mature dendritic cell (mDC) costimulatory profile, functioning as a local inflammatory adjuvant when injected into an allogeneic recipient. Intradermal DCP-001 vaccination has been shown to be safe and feasible as a post-remission therapy in acute myeloid leukemia. In the current study, the mode of action of DCP-001 was further characterized by static and dynamic analysis of the interaction between labelled DCP-001 and host antigen-presenting cells (APCs). Direct cell–cell interactions and uptake of DCP-001 cellular content by APCs were shown to depend on DCP-001 cell surface expression of calreticulin and phosphatidylserine, while blockade of CD47 enhanced the process. Injection of DCP-001 in an ex vivo human skin model led to its uptake by activated skin-emigrating DCs. These data suggest that, following intradermal DCP-001 vaccination, local and recruited host APCs capture tumor-associated antigens from the vaccine, become activated and migrate to the draining lymph nodes to subsequently (re)activate tumor-reactive T-cells. The improved uptake of DCP-001 by blocking CD47 rationalizes the possible combination of DCP-001 vaccination with CD47 blocking therapies.

## 1. Introduction

Therapeutic cancer vaccination, aiming to boost the anti-tumor immune response, could prevent tumor relapse when administered shortly after initial standard treatment when tumor burden is low. Current approaches in cancer vaccine development include cellular vaccines consisting of either killed cancer cells or autologous antigen-presenting cells (APCs) loaded with cancer antigens. Major challenges facing the development of these vaccines are low immunogenicity and, except for whole tumor cell-based vaccines, selection of relevant tumor-associated antigens (TAAs). Furthermore, autologous cellular vaccines often suffer from lack or shortage of patient-derived tumor cells or high variability in patient-derived APCs and a complex manufacturing process with a complicated supply chain. To overcome these hurdles the use of allogeneic cells is currently extensively studied as an alternative approach with the additional advantage of inducing a stronger immune response [1,2,3]. The need for an intact immune compartment for an optimal immune response to allogeneic DC-based vaccines facilitates the use of these vaccines in comparison to other allogeneic immunotherapies, such as allogeneic CAR-T cell therapies, which usually need prior patient lymphodepletion or immunosuppression to overcome host immune rejection and to limit graft-versus-host disease.

DCP-001 is a next generation cell-based vaccine that uniquely combines the positive features of (1) whole tumor cell vaccines, (2) mature dendritic cells (DCs) with pro-inflammatory adjuvant function in the allogeneic setting and (3) off-the-shelf products. As DCP-001 is generated from the myeloid leukemic cell line DCOne, it endogenously expresses multiple undefined and defined TAAs, such as the shared TAAs Wilms’ tumor-1 (WT-1), preferentially expressed antigen in melanoma (PRAME), receptor for hyaluronan-mediated motility (RHAMM), mucin-1 (MUC-1) and survivin, that are all proven to be valid targets for the immune system in both acute myeloid leukemia (AML) as well as several other cancer types, including solid tumors, such as ovarian cancer [4,5,6]. As DCP-001 is generated through differentiation and maturation of DCOne cells into cells with mature DC characteristics, its capacity to induce a proinflammatory immune response when injected into an allogeneic recipient is strongly improved, overcoming the intrinsic low immunogenicity of cancer cells [7]. Furthermore, as DCP-001 can be produced from a qualified working cell bank of DCOne cells as starting material in a standardized manufacturing process, without the need for additional antigen loading or genetic modification, it forms an ideal off-the-shelf product, bypassing many hurdles for autologous cell-based vaccines. A completed Phase I study (ClinicalTrials.gov ID NCT01373515) in AML patients demonstrated feasibility and safety of intradermal vaccination with DCP-001. Importantly, immunological assays confirmed that DCP-001 vaccination resulted in both cellular and humoral anti-tumor immune responses. Interestingly, besides T-cell responses against TAAs known to be expressed by DCP-001, T-cell responses against TAAs not expressed by DCP-001 itself were found to be re-activated or primed, most likely through epitope spreading. The induced multifunctional and lasting anti-tumor immune responses correlated with long-term survival [7,8]. These promising results led to the currently ongoing Phase II clinical trial in patients with AML that are in remission with persistent measurable residual disease (MRD) (ClinicalTrials.gov ID NCT03697707). Furthermore, a Phase I clinical trial in ovarian cancer patients after primary treatment (ClinicalTrials.gov ID NCT04739527) has been initiated recently.

From previously tested autologous DC-based vaccines it is known that the vast majority of intradermally injected cells do not migrate to the draining lymph nodes (LNs), where antigen-specific priming takes place, but remain at the injection site [9]. Furthermore, although peptide-pulsed autologous DCs lead to potent antigen-specific T-cells in vitro, the antigen-specific cytotoxic T-lymphocyte (CTL) response in vivo appears to be suboptimal, which was shown to be related to the minor role of direct T-cell activation by these DCs in vivo [10]. The same study demonstrated that the antigen-specific T-cell response induced after injection of DCs endogenously expressing the OVA model antigen was fully dependent on host DCs, suggesting an essential role of indirect antigen presentation by these cells, with so-called cross-presentation of antigens in the context of MHC class I. Such indirect antigen presentation also supports the use of allogeneic DCs as a vaccine, as no HLA-match of DC vaccine and patient is required. This is in line with the results of the DCP-001 Phase I study, where no correlations were found between degree of HLA match (between DCP-001 and patient) and observed immune responses [7]. If and how endogenous TAAs from DCP-001 are transferred to host APCs is still unclear. Various mechanisms of internalization of antigens by APCs are known, including phagocytosis, (clathrin-dependent) receptor-mediated endocytosis, non-receptor mediated endocytosis and caveolar endocytosis [11,12]. Besides these processes, the role of exosomes derived from tumor cells as transport vesicles [13] or derived from DCs themselves as antigen-presenting vesicles [14] have been described. Previous studies on interaction between DCP-001 and peripheral blood mononuclear cells (PBMCs) from multiple myeloma patients suggested a role for extracellular vesicles in antigen transfer [5], though a role of direct cell–cell contact could not be excluded.

To further elucidate the mode of action of DCP-001, we studied its interaction with allogeneic immune cells, representing host immune cells, in an in vitro setting. In particular, differentiation and maturation of leukemic DCOne cells to DCP-001 caused the induction of a strong pro-inflammatory response and increased uptake of DCP-001-derived material by allogeneic APCs in co-culture experiments. Static and dynamic analysis of the interaction between DCP-001 and allogeneic APC at single cell level clearly showed direct cell–cell interaction. More detailed studies on the cellular mechanisms underlying the uptake of DCP-001 by APCs revealed macropinocytosis, the formation of exosomes and phagocytosis as possible processes involved in this uptake. In particular, “eat-me” signals phosphatidylserine and calreticulin were found to play an important role in the process of uptake. Furthermore, inhibition of the “don’t-eat-me” signal provided by CD47 could further improve uptake of DCP-001. Finally, by use of an ex vivo human skin model, it was confirmed that DCP-001, after intradermal injection, was taken up by migrating DCs simultaneously causing activation of these cells.

## 2. Materials and Methods

### 2.1. Reagents

PBMC culture medium (MEM-α from Thermo Fisher Scientific, Waltham, MA, USA) and medium used for culture of skin biopsies (IMDM from Thermo Fisher Scientific), were supplemented with 1% pen/strep (Thermo Fisher Scientific) and 5% human pooled serum (Sigma-Aldrich, St Louis, MO, USA). For uptake assays IMDM with 1% pen/strep and 10% FBS (Thermo Fisher Scientific) was used. Phytohemagglutinin (PHA) was purchased from Thermo Fisher Scientific. Violet proliferation dye 450 (VPD450) and annexin V were from BD Biosciences (Franklin Lakes, NJ, USA). Carboxyfluorescein succinimidyl ester (CFSE), cytochalasin D, dimethyl amiloride (DMA), polyinosinic acid (PolyI), nystatin and apyrase were purchased from Merck (Darmstadt, Germany). Table S1 lists the source and catalogue numbers of the antibodies used.

### 2.2. Generation of DCP-001

DCP-001 was generated following previously described protocols [7]. In brief, cells from the myeloid leukemic cell line DCOne were cultured in a cocktail of granulocyte-macrophage colony-stimulating factor (GM-CSF), tumor necrosis factor (TNF)α and interleukin (IL)-4 in the presence of mitoxantrone to accelerate differentiation towards a DC phenotype, followed by maturation in the presence of prostaglandin-E2, TNFα and IL-1β.

### 2.3. Gamma Irradiation

DCP-001 and DCOne cells were exposed to gamma irradiation (Gammacell Elite 1000, Nordion, Ottawa, ON, Canada) at 100 Gray prior to use in all experiments, unless indicated differently.

### 2.4. Mixed Leukocyte Reaction (MLR)

DCP-001, DCOne or skin biopsy crawl-out cells, including skin-emigrated DC subsets, were seeded at various numbers of cells/well, or PHA (1% *v*/*v*) in PBMC culture medium, each in triplicate (or more, as indicated), in 96-well round-bottom culture plates (Corning, Wiesbaden, Germany). Peripheral blood lymphocytes (PBLs) from healthy donors were labelled with 2 μM CFSE for 10 min at 37 °C, and 1.0 × 10^5^ labelled PBLs in PBMC culture medium were added to each well. After 6 days of culture at 37 °C, proliferation of PBLs was measured by flow cytometry, corrected for background proliferation and normalized to the proliferation by PHA (set at 100%).

### 2.5. Peripheral Blood Mononuclear Cell (PBMC) Stimulation Assay

DCOne or DCP-001 cells were co-cultured with allogeneic PBMCs from a healthy donor at a 1:1 ratio, in triplicate, in 96-well round-bottom culture plates in PBMC culture medium. After 6 days of culture at 37 °C, supernatants were harvested and stored at −20 °C until further use.

### 2.6. Cytokine/Chemokine Analysis Employing the Luminex Platform

Multianalyte profiling of cytokines and chemokines was performed using the Luminex MAGPIX^®^ system (Luminex, Austin, TX, USA). Supernatants from PBMC stimulation assays were tested by use of customized 8- and 3-plex kits provided byThermo Fisher Scientific, whereas 24 h supernatants from uptake assays were tested using various customized multiplex kits from Bio-Techne (Minneapolis, MN, USA) (see Table S1). All analyses, including sample activation for measurement of TGFβ-1, -2 and -3, were performed according to the manufacturers’ protocols. Acquired fluorescence data were analyzed by the 4.3 ×PONENT software (Luminex).

### 2.7. Isolation of PBMCs and Generation of Immature Monocyte-Derived DCs (imoDCs)

PBMCs were isolated from human healthy volunteers (Sanquin, Amsterdam, The Netherlands) using gradient density centrifugation over Lymphoprep (Stemcell Technologies, Vancouver, BC, Canada). Monocytes were isolated from PBMCs according to the manufacturer’s instructions using an EasySep™ Human Monocyte Enrichment Kit (Stemcell Technologies). A total of 5–6 × 10^6^ monocytes were cultured in one T75 cell culture flask (Greiner Bio-One, Kremsmünster, Austria) using IMDM containing 10% FBS, 100 μg/mL penicillin and 100 μg/mL streptomycin (all from Thermo Fisher Scientific) and supplemented with 1000 IU/mL GM-CSF and 10 ng/mL IL-4 (Miltenyi, Bergish Gladbach, Germany). After 6–7 days, imoDCs were collected and used.

### 2.8. Uptake Assay and Blocking of Uptake

PBMCs or imoDCs were labelled with 1 µM VPD450, and DCP-001 or DCOne cells were stained with 2 µM CFSE, both for 10 min at 37 °C. After washing, VPD450-labelled PBMCs or imoDCs were co-cultured 1:1 with CFSE-labelled DCP-001 or DCOne cells in a 24- or 48-well cell culture plate (Corning) at 37 °C. In some uptake experiments, blocking agents or antibodies were added during co-culture. For the co-cultures with blocking antibodies, except for anti-CD47 and anti-SIRPα, an additional 30 min pre-incubation at 4 °C or room temperature of host imoDCs with the blocking antibodies or relevant isotype control antibodies took place. As the antibodies specific to CD47 and SIPRα contained azide, blocking of CD47 on DCP-001 or SIPRα on imoDCs was performed by a 30 min pre-incubation of DCP-001 with anti-CD47 (or relevant isotype control antibody including azide) or imoDCs with anti-SIRPα (or relevant isotype control antibody including azide), respectively, after which the cells were washed prior to co-culture. After 4 h co-culture, cells were harvested and stained for anti-CD274-APC for 30 min at 4 °C. Uptake was evaluated as the percentage of the CFSE/VPD450-positive population in the APC-positive PBMCs/imoDCs using a FACSVerse^TM^ flow cytometer (BD Biosciences).

### 2.9. Confocal Microscopy

ImoDCs and DCP-001, labelled with VPD450 and CFSE, respectively, as described for the uptake assay, were cultured in a 1:1 ratio on a µ-Slide 8 Well chamber slide (ibidi, Gräfelfing, Germany) for 4 h. Cells were then fixed in 4% paraformaldehyde for 10 min at 4 °C. Rhodamin pallodine (1:400, Thermo Fisher Scientific) was added to the cells for further incubation at room temperature in the dark for 2 h. For imaging of the skin crawl-out cells, cells were additionally counterstained with DAPI 1:10,000 (Agilent Technologies, Santa Clara, CA, USA) for 10 min. After washing with PBS once, images were captured using a confocal microscope (Leica SP8 STED, Leica microsystems, Wetzlar, Germany). Fluorescence intensity was measured using ImageJ software version 1.8.0 (NIH, Bethesda, MD, USA).

### 2.10. Dynamic Single-Cell Analysis by Droplet Array-Based Microfluidic Platform

The dynamic interaction between DCP-001 and imoDCs was assessed at a single-cell level by use of a droplet array-based microfluidic platform, as described by Sarkar et al. [15]. Briefly, CSFE-labelled DCP-001 and CMTPX-labelled imoDCs were co-encapsulated in microdroplets at a 1:1 ratio. During a total time of 4 h, the mobility of cells and their interactions were recorded.

### 2.11. Phenotypic Analysis

Phenotypic analysis of DCOne and DCP-001 cells (1 × 10^5^ each) was performed by single staining with fluorochrome-conjugated CD-marker specific antibodies or their relevant isotype controls (indicated in Table S1) for 30 to 60 min at 4 °C. Skin biopsy crawl-out cells were (co)stained with fluorochrome-conjugated antibodies or relevant isotype controls (indicated in Table S1) for 30 to 60 min at 4 °C. Stained cells were analyzed by flow cytometry using FlowJo software version 10.1 (BD Biosciences).

### 2.12. Human Skin Explant Culture and Analysis of Migrating Cells

Human abdominal skin (Tergooi Hospital, Hilversum, The Netherlands) was obtained within 24 h following abdominal resection of healthy donors after informed consent and used for intradermal injection in explants as described previously [16,17] with minor modifications. Briefly, Terumo (Tokyo, Japan) needles (21G, 1½” × 0.8 × 40 mm) (representative for the needles used to inject DCP-001 during vaccination of patients) were used to intradermally inject 25 μL of serum-free medium with or without GM-CSF (1000 IU/mL)/IL4 (10 ng/mL) or with 5 × 10^5^ CFSE-labelled DCP-001 cells, so a small blister appeared. CFSE labelling was performed as described above in the uptake assay. A punch biopsy (8 mm;Avantor, Radnor Township, PA, USA) surrounding the blister was taken, and 48 to 106 biopsies per condition were cultured with the epidermis facing upwards in a 48-well plate with 1 mL medium/well for 72 h. Biopsies were discarded and crawl-out cells harvested and pooled per condition prior to phenotypic analysis or use in MLR.

### 2.13. Statistics

Data were analyzed using GraphPad Prism (GraphPad Software, Inc., San Diego, CA, USA) and expressed as mean ± SEM or as indicated. At least three independent experiments were conducted for each treatment, except for the skin explant experiment which was performed twice. The Shapiro–Wilk test was used to determine normal data distribution. Statistical significance was determined by paired or unpaired Student’s *t*-test. *p* < 0.05 was considered to be statistically significant.

## 3. Results

### 3.1. The Shift towards a Mature DC Phenotype Causes DCP-001 to Induce a Strong Pro-Inflammatory Response When Co-Cultured wth Allogeneic Lymphocytes

**Figure 1:**
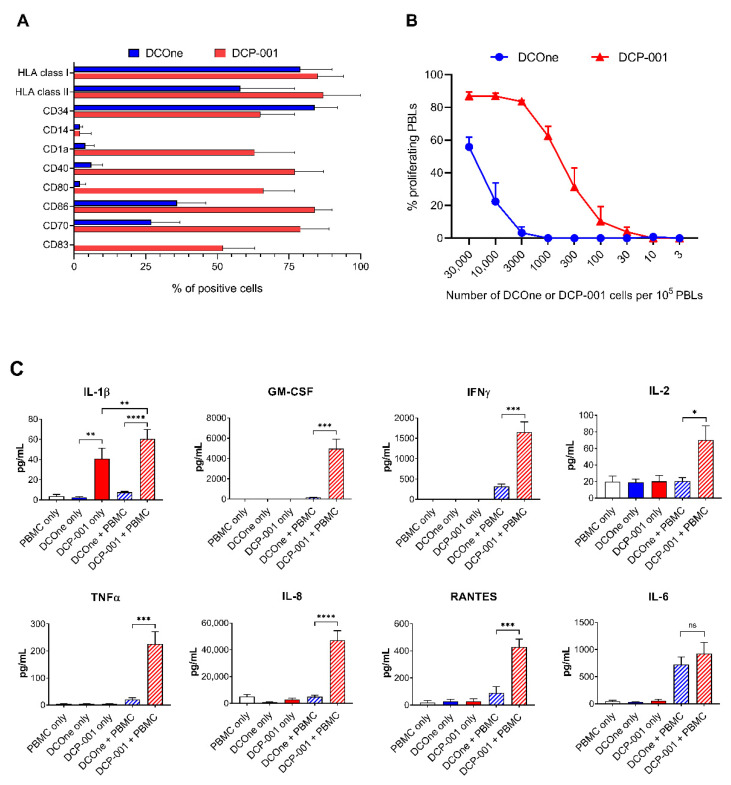
High immunogenicity of DCP-001. (**A**) The phenotypic characteristics of DCP-001 (*n* = 672 for all markers except CD70: *n* = 116) and DCOne (*n* = 57) were determined by flow cytometry. All indicated CD markers were expressed at significantly higher percentages of cells in DCP-001 than in DCOne, except for CD34, which was expressed by significantly more cells in DCOne than in DCP-001, and for CD14, which was expressed by non-significantly different percentages of cells in DCOne and DCP-001. (**B**) The PBL stimulatory activity of DCP-001 and DCOne was tested in an MLR assay (four independent experiments). As a positive control, PHA was included and used for normalization of the results (PHA proliferation set at 100%). (**C**) DCP-001 and DCOne were tested in a PBMC stimulation assay by co-culture for 6 days after which supernatants were collected for multiplex analysis on a Luminex platform. Data represent 12 independent experiments. Data are expressed as mean ± SEM. ns = nonsignificant; * *p* < 0.05; ** *p* < 0.01 *** *p* < 0.001; **** *p* < 0.0001 significant difference between indicated groups determined by paired *t*-test analysis.

To eliminate any proliferative capacity, DCOne and DCP-001 were gamma-irradiated prior to all experiments. As shown in Figure 1A, compared to DCOne, DCP-001 showed significantly increased percentages of cells expressing CD1a, CD40, CD70, CD80, CD86, CD83, HLA class I and HLA class II, while both DCOne and DCP-001 showed low percentages of cells expressing CD14 and high percentages of cells expressing CD34. Accordingly, DCP-001 induced a stronger proliferative response in allogeneic PBLs (Figure 1B) and, in allogeneic PBMCs, induced release of pro-inflammatory cytokines and chemokines, including type-1 T-cell cytokines, at levels far exceeding those induced by DCOne (Figure 1C). Interestingly, DCP-001 itself released IL-1β, which was not observed for DCOne. These results clearly demonstrate that DCP-001′s mature DC phenotype leads to the capacity of the vaccine to induce a strong pro-inflammatory immune response in the allogeneic setting.

### 3.2. Cellular Content of DCP-001 Is Efficiently Captured by Host APCs

**Figure 2:**
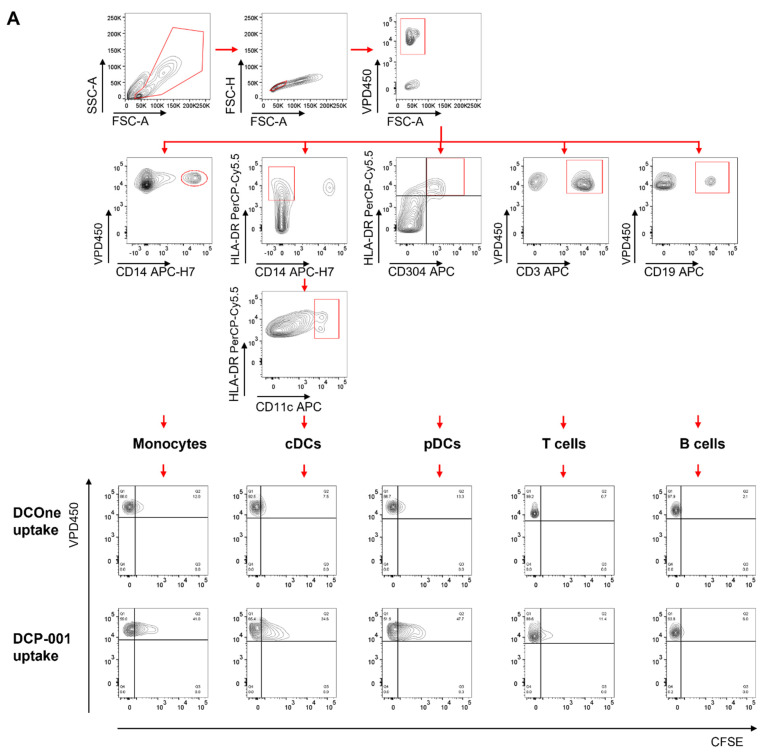

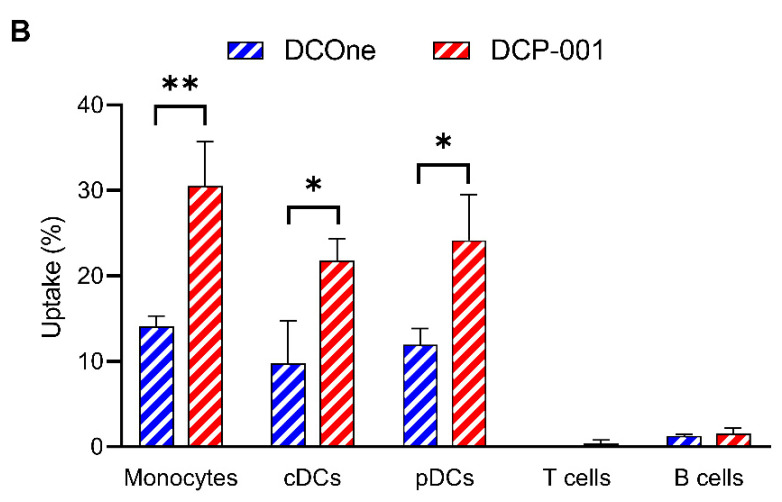
DCP-001 is taken up by antigen-presenting cells in PBMCs. In uptake experiments VPD450-labelled PBMCs were co-cultured with CFSE-labelled DCP-001 or DCOne for 4 h. (**A**) Representative gating strategy to identify uptake of CFSE-labelled DCP-001 or DCOne by different cell populations. Upper panel: first, from the FSC-SSC plot, lymphocytes were gated, then from the FSC height-FSC area plot single cells were gated, and from these cells VDP450-labelled cells were gated. Middle panel: these gated cells were further identified as either monocytes (CD14^+^), myeloid DCs (CD11c^hi^, HLA-DR^+^, CD14^−^), plasmacytoid DCs (CD304^+^, HLA-DR^+^), T-cells (CD3^+^) or B-cells (CD19^+^). Lower panel: finally, uptake was assessed by the CFSE-positive population in each of the subpopulations. FSC, forward scatter; SSC, side scatter. (**B**) Uptake of DCP-001 or DCOne was determined by the percentage of VPD450/CFSE-positive cells in each of the specific PBMC subpopulations. The uptake was corrected for any non-specific uptake at 4 °C. Data represent three independent experiments. Data are expressed as mean ± SEM, * *p* < 0.05, ** *p* < 0.01; significant difference between indicated groups, using multiple t-test with Holm–Sidak method.

To investigate whether DCP-001 is captured by host immune cells, DCP-001 and DCOne were labelled with CFSE and co-incubated with VPD450-labelled PBMCs. Thereafter, the percentage of CFSE/VPD450 double-positive cells among monocytes, myeloid DCs, plasmacytoid DCs, B-cells and T-cells was determined as shown in Figure 2A,B. Cell subsets that were most efficient in capturing DCP-001-derived material were professional APCs, including monocytes and DCs (both conventional, cDC, and plasmacytoid DC, pDC, all showing around 20% to 30% uptake). Cellular content of DCP-001 was captured by these cells to a significantly higher extent than that of the original leukemic DCOne cells, indicating that the mature DC phenotype of DCP-001 increases its ability to bind to and be captured by APCs.

**Figure 3:**
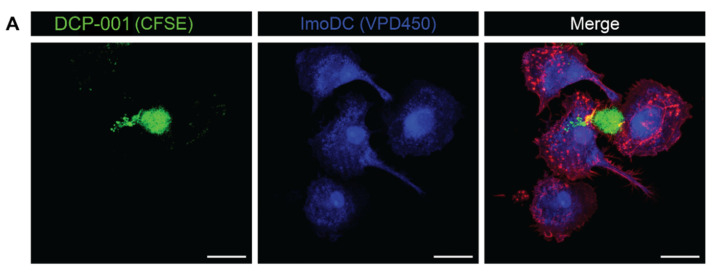

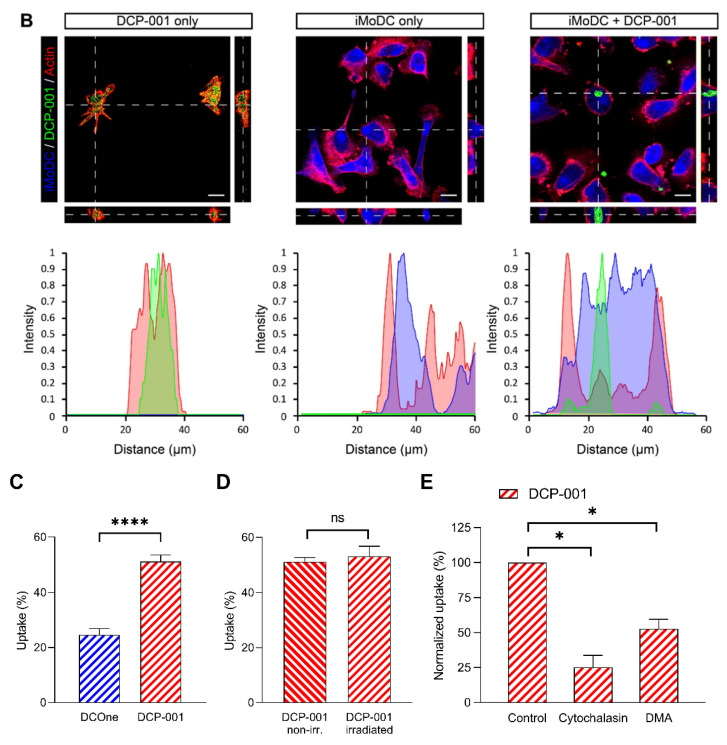
Host imoDCs are able to internalize DCP-001 after in vitro co-culture for 4 h by a process of endocytosis. CFSE-labelled DCOne-derived cells (green) were co-incubated with VPD450 stained imoDCs (blue) in a 1:1 ratio for 4 h at 37 °C. (**A**) Confocal images indicated the direct interaction and internalization of DCP-001 by host imoDCs. Actin (cytoskeleton) was stained by rhodamine-labelled phalloidin (red). Scale bar: 20 µm. (**B**) Z-stack images were further analyzed using ImageJ software. Overlays of histograms, each for the intensity of a specific fluorochrome, were created for a selected area (indicated by dashed lines) (**C**) After the 4 h of co-culture, cells were stained for APC-conjugated anti-CD274 for 30 min at 4 °C. The percentage uptake was determined as the VPD450/CFSE double positive population in the total APC-positive imoDC population. Data represent 16–25 independent experiments. (**D**) The uptake of irradiated DCP-001 (as used in all experiments) was not different from the uptake of non-irradiated DCP-001. Data represent three independent experiments. (**E**) The uptake of DCP-001 was blocked by the addition of cytochalasin (10 µM) and DMA (500 µM) during the co-culture. The control uptake (uptake without blocking agents) was set at 100% for normalization of the data. Data represent three independent experiments. Data in (**C**) to (**E**) are expressed as mean ± SEM; * *p* < 0.05; **** *p* < 0.0001 significant difference; ns = non-significant difference between indicated groups.

The uptake of DCP-001 by imoDCs was visualized by confocal microscopy (Figure 3A). DCP-001 attached to the imoDCs and (CFSE-labelled) DCP-001 material was engulfed by these DCs at actin-enriched membrane–membrane contact points, confirming cell–cell contact for uptake. To confirm that DCP-001-derived material was indeed localized inside imoDCs, histograms for signal intensity of each dye are shown in orthogonal cross sections of the images as shown in Figure 3B.

Besides this static cell–cell interaction assessment, dynamic interactions between DCP-001 and imoDCs were assessed at a single cell level by use of a droplet array-based microfluidic platform. During 4 h, the mobility of cells and their interactions were recorded. A video (Video S1) clearly demonstrates active DCP-001/imoDC interactions that could result in cell-associated antigen transfer from DCP-001 to imoDCs.

### 3.3. Macropinocytosis and/or Phagocytosis Contributes to the Uptake of DCP-001 by APCs

Endocytic processes such as phagocytosis and macropinocytosis, and membrane extrusions needed for formation of “synapses” and exosomes, are accompanied by membrane ruffling and actin polymerization. DMA and cytochalasin-D affect actin polymerization, and nystatin is a specific inhibitor of caveolar endocytosis. These three agents were tested during uptake of DCP-001 by imoDCs to elucidate whether these endocytic processes might play a role. To ascertain whether these processes could also underlie a more efficient uptake of DCP-001 over that of DCOne, both were included in these experiments. Similar to the peripheral-blood-derived DCs, the imoDCs were able to efficiently capture DCP-001 (50%) and again at a level that was about 2-fold higher than the capture of DCOne (Figure 3C). As gamma irradiation might affect the efficiency of uptake, the uptake of DCP-001 batches, each split by being irradiated or not, was tested. However, no effect of irradiation on uptake was observed (Figure 3D). When DMA or cytochalasin was added during the co-culture of DCP-001 and imoDC, uptake of DCP-001 was significantly reduced by about 80% or 50%, respectively (Figure 3E), with similar effects in co-cultures with DCOne. Nystatin did not affect uptake at all (data not shown). These results indicate a major role for endocytosis-mediated internalization of DCP-001-derived material by imoDCs, with phagocytosis and/or macropinocytosis as the most important processes of endocytosis.

### 3.4. “Eat-Me” Signals Calreticulin and Phosphatidylserine and “don’t-Eat-Me” Signal CD47 Are Key Factors in the Efficient Endocytosis of DCP-001-Derived Material

**Figure 4:**
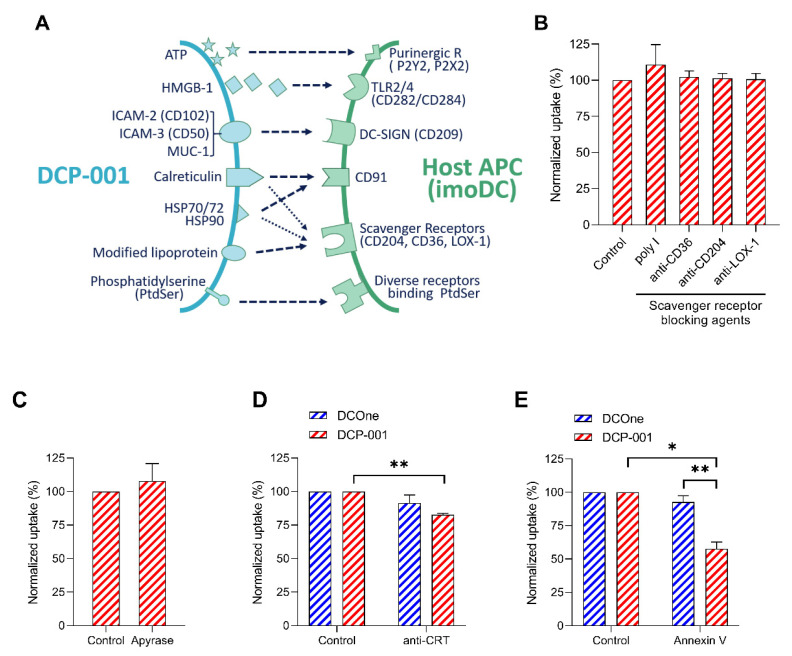

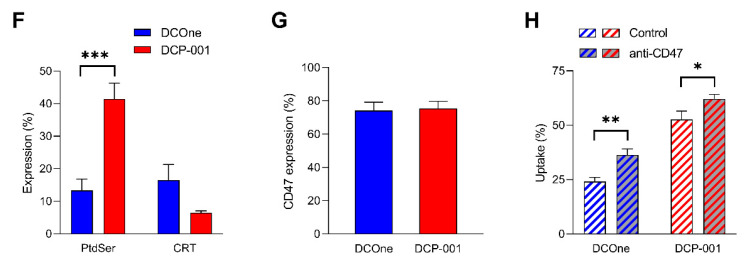
“Eat-me” and “don’t-eat-me” signaling molecules CRT, PtdSer and CD47 play a vital role in the uptake of DCP-001. (**A**) Schematic diagram showing potential receptor/ligand interactions that may contribute to the uptake of DCP-001 by host APCs (imoDCs). (**B**) To evaluate the role of scavenger receptors in uptake of DCP-001 by host imoDCs, polyinosinic acid (50 µg/mL), anti-CD36 antibody (50 µg/mL), anti-CD204 antibody (50 µg/mL) and anti-LOX-1 antibody (50 µg/mL) were added during the uptake assay. Data are expressed as percentage uptake normalized to control (uptake without any blocking agent or with the relevant isotype control, set at 100%) and as mean of three independent experiments ± SEM. (**C**–**E**) Apyrase, CRT-specific antibody and purified recombinant annexin V were added in the uptake experiments to investigate the role of ATP, CRT and PtdSer, respectively. Data are expressed as percentage uptake normalized to control (uptake without any blocking agent or with the relevant isotype control, set at 100%) and as mean of three to four independent experiments ± SEM. (**F**,**G**) The expression of PtdSer (see also Figure 5), CRT and CD47 on the surface of DCOne cell and DCP-001 was determined by flow cytometry. Data are presented as mean ± SEM of three to six batches of DCOne and DCP-001. (**H**) Monoclonal antibody specifically targeting CD47 was used to investigate the role of CD47 in preventing uptake of DCOne or DCP-001, using the relevant isotype antibody as control. Data are expressed as mean ± SEM of three to four independent experiments, * *p* < 0.05, ** *p* < 0.01, *** *p*< 0.001 significant difference between indicated groups.

An array of cell surface receptor–ligand pairs, including scavenger receptors and danger-associated molecular pattern (DAMP) receptors may potentially play a role in the interaction between DCP-001 and APCs, leading to endocytosis (Figure 4A).

To investigate the role of scavenger receptors, monoclonal antibodies to scavenger receptor A (CD204), scavenger receptor B (CD36) and scavenger receptor lectin-type oxidized LDL receptor (LOX-1) were added to a co-culture of DCP-001 and imoDCs. In addition, the effect of competitively inhibiting scavenger receptor ligand polyinosinic acid (poly(I)) was tested. None of these blocking agents affected uptake of material from either DCP-001 (Figure 4B) or DCOne (latter not shown).

**Figure 5:**
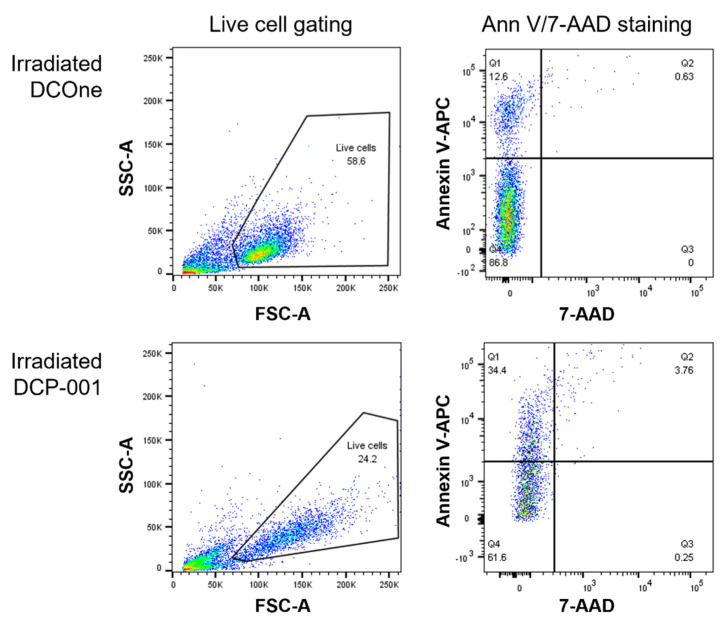

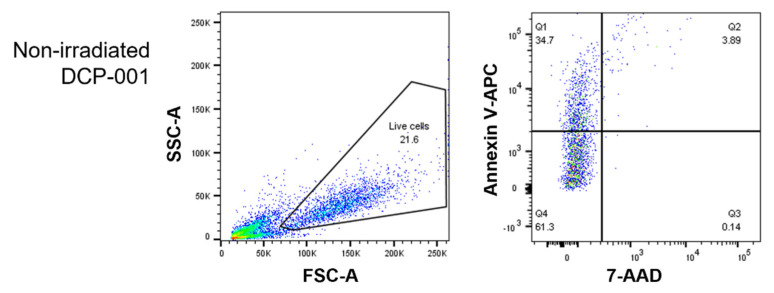
Increased cell surface expression of PtdSer on viable DCP-001 cells is not induced by irradiation. Irradiated DCOne, irradiated DCP-001 and non-irradiated DCP-001 were cultured for 4 h at 37 °C (same conditions as during the uptake assay). Cells were then washed and resuspended in annexin V staining buffer. Annexin V-APC and 7-AAD were added, and cells were incubated for another 15 min at room temperature in the dark. Flow cytometric analysis was performed on the, in the FSC-A/SSC-A plot, gated live cell population (see left column). The percentage annexin V-APC-positive/7-AAD-negative cells (Q1) (see right column) represented the % PtdSer-positive cells as shown in Figure 4F.

In a subsequent series of uptake experiments, monoclonal antibodies against CD91, CD102, CD209, CD282 and CD284 were each tested as inhibitory agents, but none of these antibodies resulted in any inhibition of uptake (data not shown). To assess whether ATP release by DCP-001 or DCOne could play a role in the uptake, cells were incubated with apyrase, which catalyzes the hydrolysis of ATP. This treatment also did not affect uptake (Figure 4C). However, when the “eat-me” signals calreticulin (CRT) or phosphatidylserine (PtdSer) were blocked by use of a calreticulin-specific antibody or annexin V, respectively, a significant reduction in uptake was observed, with a percentage inhibition of uptake of DCP-001 significantly exceeding that of DCOne (Figure 4D,E). Indeed, surface expression of PtdSer was found to be significantly increased on DCP-001 in comparison to DCOne (Figure 4F), in keeping with a superior blocking effect of annexin V. Again to determine whether this increased exposure of PtdSer could have been induced by irradiation, non-irradiated DCP-001 was also tested. In addition, for all PtdSer expression experiments, cells were co-incubated with the live/dead staining 7-amino-actinomycin D (7-AAD) to exclude PtsSer on dead or late apoptotic cells. The flow cytometric data are shown in Figure 5, showing no difference in the percentage irradiated versus non-irradiated DCP-001 cells expressing PtdSer on their surface. The MFI of the PtdSer staining for these two conditions was also not different, though it was about 2 times lower on irradiated DCOne cells.

Finally, the role of “don’t-eat-me” signal CD47 on DCOne and DCP-001 in their uptake by imoDCs was investigated. CD47 is abundantly overexpressed on several types of cancer cells (especially cancer stem cells) representing a potent strategy for immune evasion [18]. Indeed, expression of this molecule was found on about 75% of the DCOne cells. Equivalent percentages of cells in DCP-001 expressed CD47 (Figure 4G) at similar levels per cell (MFI data, not shown), yet when uptake by imoDCs was tested after pre-incubation with anti-CD47 antibody, a more pronounced increase in uptake of DCOne versus DCP-001 was observed (Figure 4H), indicating that CD47-signalling is indeed a mechanism through which material derived from the leukemic DCOne cells is less well captured by imoDCs. Nevertheless, blockade of CD47 was still capable to further improve the uptake of DCP-001. Preliminary data of blocking of SIRPα, the receptor of CD47, on the imoDC site resulted in a similar increase in DCP-001 uptake (data not shown).

### 3.5. DCP-001 Induces Phenotypic and Functional Maturation of Co-Cultured Allogeneic DCs

**Figure 6:**
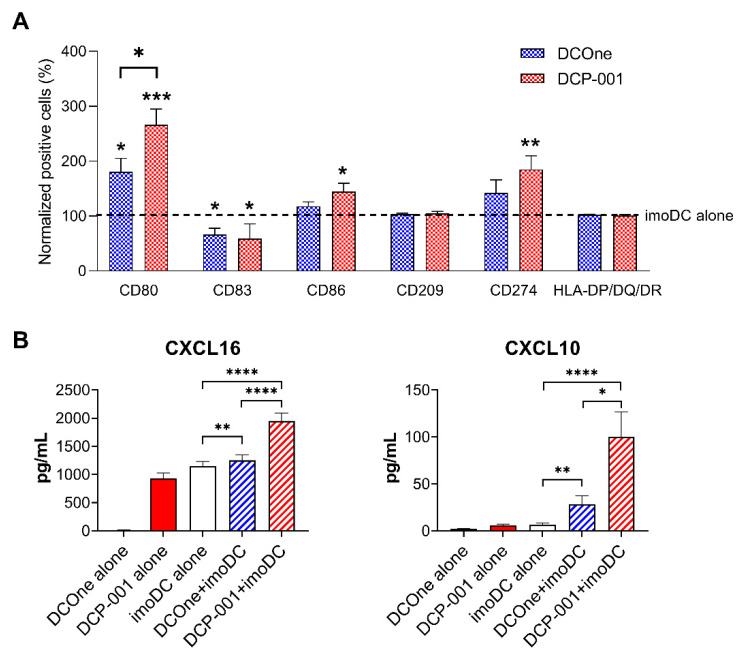
The maturation of imoDCs and release of cytokines/chemokines after 24 h of in vitro co-culture. (**A**) After 24 h of in vitro co-culture, the phenotype of allogeneic imoDCs was determined for percentage cells expressing CD80, CD83, CD86, CD209, CD274 and HLA-DP, -DQ and -DR using flow cytometry. Percentage of cells stained by these markers among imoDC cultured without DCP-001 or DCOne was set at 100% for normalization of the data. Data represent five to six independent experiments. Stars above the bars indicate significant difference to imoDC alone. (**B**) Levels of CXCL10 and CXCL16 in the cell culture supernatant collected 24 h after in vitro co-culture were measured using the Luminex platform. Data represent six independent experiments. Data are expressed as mean ± SEM, * *p* < 0.05, ** *p* < 0.01, ***p < 0.001, **** *p* < 0.0001 significant difference between indicated groups.

To evaluate the effect of the interaction between DCP-001 and allogeneic imoDC, including DCP-001 uptake, the phenotype of imoDCs cultured for 24 h in the presence or absence of DCP-001 or DCOne was analyzed. Key maturation markers (CD80, CD83 and CD86), antigen-presenting molecules (HLA-DP,-DQ and -DR) and molecules that influence T-cell activation (PD-L1 [CD274] and DC-SIGN [CD209]) were included in the phenotypic analysis. Results are shown in Figure 6A. DCP-001 significantly induced expression of CD80, CD86 and PD-L1 on imoDCs, while DCOne only induced CD80 expression but to a lower degree. In contrast, CD83 levels decreased marginally, while HLA-DR and DC-SIGN levels were unaffected. These data thus indicate that interaction between DCP-001 and allogeneic DCs, including uptake of DCP-001, increases the maturation state of the allogeneic DCs.

In line with their increased phenotypic maturation, the co-cultured allogeneic DCs were found to release significantly enhanced levels of CXCL10 and CXCL16 upon uptake of DCP-001 (Figure 6B). These chemokines are known to be expressed by activated DCs and are involved in the recruitment of T- and NK cells to effector sites and T-cell areas of secondary and tertiary lymphoid structures, respectively. In contrast, no cytokines were detected with tolerizing properties, e.g., IL-10 and TGFβ.

### 3.6. Uptake of DCP-001 by Skin-Emigrated DC Subsets

**Figure 7:**
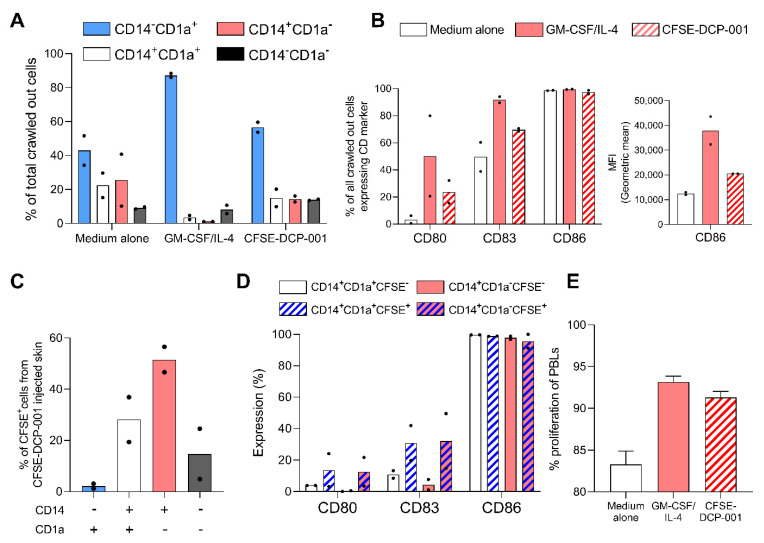

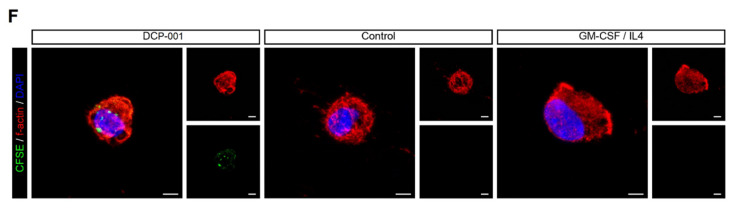
Antigen-presenting cells that have taken up DCP-001 migrate out of skin tissue and display an activated phenotype. Human abdominal skin explants were used for intradermal injection of medium alone (negative control), medium containing GM-CSF/IL-4 (positive control) or medium containing CFSE-labelled DCP-001 (CFSE-DCP-001). Punch biopsies surrounding the injection sites were taken and placed in culture medium for 72 h. Crawl-out cells were harvested; co-stained with CD34-PerCP-Cys5.5, CD1a-APC, CD14-APC-fire, CD80-BV510, CD86-PE-Cy7 and CD83-BV421 and analyzed by flow cytometry. Data shown in A-D are from two experiments with dots as individual and bars as mean results. (**A**) Percentage of crawl-out cells stained for CD1a/CD14/CD34 in each condition, where CD34 was negative for all. (**B**) Percentage of crawl-out cells stained for CD80, CD83 and CD86 (**left**) and mean fluorescence intensity (MFI) for CD86 (**right**) in each condition. MFIs for other markers (not shown) did not differ between conditions. (**C**) Percentage of CD1a/CD14 stained crawl-out cells positive for CFSE in the condition where CFSE-labelled DCP-001 was injected. (**D**) Percentage of CD14^+^ crawl-out cells that have taken up DCP-001 (CFSE^+^) or not (CFSE^−^) stained for CD80, CD83 and CD86 in the condition where CFSE-labelled DCP-001 was injected. MFIs of markers did not differ between conditions (data not shown). (**E**) Proliferative response of PBLs from a healthy individual, co-cultured for 6 days with crawl-out cells from each condition at a PBL/crawl-out cell ratio of 10:1. Data shown are from one experiment, with results normalized to PHA as positive control; mean + SD of 4-fold (medium alone) or 6-fold replicate measurements. (**F**) Confocal images of a craw-out cell in each condition. Actin (cytoskeleton) was stained by rhodamine-labelled phalloidin (red), nuclei are stained with DAPI (blue) and CFSE, derived from CFSE-labelled DCP-001, is stained green. Scale bar: 20 µm.

To explore whether antigen transfer from DCP-001 to tissue-resident DC subsets might occur after intradermal injection, an ex vivo human skin explant model was used. CFSE-labelled DCP-001 was intradermally injected into healthy human skin tissue, whereupon biopsies covering the injection site were taken and cultured in medium for 3 days. As negative and positive controls for the activation of subsequently migrating DCs, injections of medium alone and medium containing GM-CSF/IL-4, respectively, were included. Cells migrating out of the skin biopsies were harvested and analyzed for their phenotype and CFSE content. As shown in Figure 7A the majority of cells migrating out of the skin were CD1a^+^, representing Langerhans cells and dermal DCs. The fact that these cells were negative for CD34 excluded the possibility that these cells were derived from DCP-001 itself, since these cells are known to be highly CD34-positive. The maturation status of the crawl-out cells from skin injected with DCP-001 compared to negative control was clearly increased as shown by increased percentages of CD80- and CD83-expressing cells and increased MFI of CD86 (Figure 7B). From all CD1a^+^ cells migrated out of the skin injected with CFSE-labelled DCP-001, 30% were CFSE-positive. Most of these cells were also CD14^+^, whereas about 50% of the CD1a^−^/CD14^+^ cells (representing macrophage-like dendritic cells) were CFSE-positive, indicating efficient uptake of DCP-001 by these APCs in particular (Figure 7C). All subsets that had taken up CFSE-labelled DCP-001 material carried higher surface levels of activation markers as compared to their phenotypic counterparts that had not taken up any CFSE-labelled DCP-001 material (Figure 7D). Finally, crawl-out cells from skin injected with DCP-001 showed an increased T-cell stimulatory capacity as demonstrated by an MLR (Figure 7E). Confocal microscopic images confirm the ingestion of DCP-001 material by skin-emigrated dendritic cells (Figure 7F).

These data strongly suggest that DCP-001 after intradermal injection in patients, rather than migrating to draining lymph nodes itself, is ingested by skin-derived APCs, that are subsequently able to migrate and are activated by the uptake of DCP-001, resulting in an increased T-cell priming/reactivation ability. This latter aspect will be further investigated in future studies.

## 4. Discussion

DCP-001 is an allogeneic cell-based vaccine differentiated from the DCOne cell line and expresses the mature DC costimulatory machinery. Data presented in this study indicate that the most likely mode of action of DCP-001 vaccination, which leads to anti-tumor T-cell responses as shown in the clinical setting [7], entails enhanced activation of skin-resident alloreactive T-cells, causing local inflammation and transfer of TAAs from DCP-001 to attracted host DCs, which in turn migrate to draining lymph nodes to initiate the tumor-specific T-cell priming process.

As DCP-001 originates from a myeloid leukemia cell line, it expresses a broad range of TAAs, some of which are expressed by other types of hematological malignancies [4,5,6,19] as well as by solid tumors (e.g., ovarian cancer) [6,20]. In addition, DCP-001 vaccination of AML patients was also shown to induce immune responses towards dominant TAAs not expressed by DCP-001 itself such as NY-ESO-1 and MAGE-A3, suggesting the spreading of the immune response induced by the vaccine towards patient tumor-specific antigens [7]. Therefore, DCP-001 is an attractive vaccine candidate for different types of cancer.

In vitro uptake experiments showed that DCP-001-derived material is captured by APCs and that efficiency of this uptake process, in comparison to that of DCOne cells, is significantly increased. In terms of antigen transfer between DCs, this makes sense as DC maturation signals danger and the need for specific T-cell activation. Inhibition of actin polymerization blocked the uptake of DCP-001, suggesting that macropinocytosis and/or phagocytosis form the underlying general processes of uptake by APCs. This type of internalization has also been described as an essential part of both the immune surveillance function as well as the MHC class I and II antigen (cross-)presenting capacity of immature DCs [12,21]. From a broad range of factors that are known to play a role in endocytosis of cells, the “eat-me” signals calreticulin and PtdSer and the “don’t-eat-me” signal CD47 were all found to contribute to uptake of DCP-001.

Interestingly, when compared to DCOne cells, DCP-001 showed significantly higher percentages of cells exposing PtdSer at the cell surface, correlating with a higher uptake of DCP-001. Furthermore, when specifically blocking PtdSer by annexin V, DCP-001 uptake was drastically reduced. Cell surface exposure of PtdSer is known to be increased when cells become apoptotic [22]. Apoptosis might also be induced by irradiation [23]. However, the level of PtdSer on DCP-001 did not increase after irradiation, and irradiated DCP-001 was equally well captured by allogeneic imoDCs as non-irradiated DCP-001. Furthermore, in all experiments, DCOne was exposed to the same strength of gamma irradiation as DCP-001 but was still consistently less well taken up by imoDCs. Therefore, a role of gamma irradiation in the efficient uptake of DCP-001 is excluded. Alternatively, the differentiation and/or maturation step to obtain DCP-001 from DCOne might either induce apoptosis as has been described for in vitro matured serum-deprived DCs [24] or cause upregulation of cell surface PtsSer as has been described for activated T-cells [25]. Uptake of cells via PtdSer has been described as “efferocytosis”, a general mechanism for maintenance of homeostasis, clearing apoptotic cells without maturation of the DCs and with induction of immune suppressive cytokines [26,27]. However, the phenotypic change of allogeneic imoDCs after uptake of DCP-001 showed increased expression of maturation markers CD80 and CD86. Expression of checkpoint inhibitory molecule PD-L1, which binds to CD80 in cis, was also increased. However, with equal increase in CD80 expression, the functional role of CD80 is not expected to be diminished [28]. Furthermore, typical immune suppressive cytokines that are released during efferocytosis, such as IL-4, IL-10 and TGFβ, could not be detected in the co-culture supernatant. Release of IL-1β and CXCL16 by DCP-001 itself, creating a pro-inflammatory milieu, is possibly driving this shift towards more mature and inflammatory DCs. On top of that, when DCP-001 is injected in the skin, additional pro-inflammatory cytokines are expected to be released by activated skin-resident alloreactive T-cells. These skin-resident alloreactive T-cells are found to be (often virus-specific) memory T-cells [29], that readily release inflammatory cytokines such as interferon (IFN)γ, TNFα and IL-2 upon rechallenge [30,31]. This creates a strong pro-inflammatory environment, driving the dermal DCs (that have taken up DCP-001 cellular content) towards migratory pro-immunogenic DCs [32]. In addition, in vitro studies showed that allogeneic T-cells stimulated with DCP-001 are polarized to a more Th1 type of response, while induction of regulatory T-cells is not observed [5].

The role of PtdSer and its many receptors in the uptake process of PtdSer-expressing cells has been subject of study for many years [33]. The current idea (at least for tissue resident macrophages) is that uptake via PtdSer starts with two steps called “tethering” and “tickling”, leading to phagocytic synapse formation as part of the macropinocytosis process [34]. Moreover PtdSer exposed on CD8+ T-cells upon their activation has been shown to lead to synapse formation with DCs [25]. Interestingly, a recent paper by Ruhland et al. [35] showed a similar type of formation of cell–cell contacts and synapses between myeloid cells in tumor-draining LNs. Furthermore, a recent study using a murine lung tumor model demonstrated the crucial role of PtdSer receptor TIM-4 in DCs for uptake and cross-presentation of tumor-associated antigens to anti-tumor CD8+ T-cells [36].

In addition to the two “eat-me-signals”, a role for “don’t-eat-me-signal” CD47 in the uptake of DCP-001 was demonstrated. Interestingly, various types of cancer seem to express high levels of CD47 to escape uptake by phagocytes [18]. No change in cell surface expression of CD47 was observed when DCOne cells were differentiated and matured into DCP-001, and blocking this molecule increased uptake of both DCOne cells and DCP-001 by imoDCs. However, more pronounced blockade of the uptake of DCOne cells was observed which might be due to stronger compensating “eat-me-signals” (PtdSer in particular) for DCP-001 versus DCOne cells. Alternatively, differences in glycosylation of CD47 might play a role in different functionality of the molecule, though additional studies would be needed to confirm this. Agents blocking the CD47-specific pathway, including CD47-specific antibodies, are currently tested as therapy alone or in combination with other therapies in different cancer types [37,38]. The positive effect of anti-CD47 in uptake of DCP-001 forms a rationale for combining DCP-001 vaccination with one of these CD47-targeting therapies.

**Figure 8:**
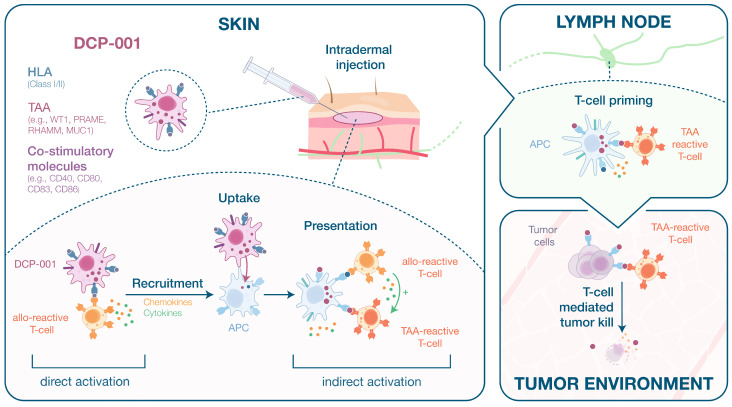
Proposed mode of action of DCP-001 (see explanatory description in the Discussion below).

The data described in this paper support the proposed mode of action of the allogeneic cell-based vaccine DCP-001, which is (re)activation of tumor-specific T-cell responses by (1) its capacity to induce a strong local pro-inflammatory allogeneic response that leads to recruitment and activation of host DCs and (2) subsequent indirect TAA presentation by lymph-node migrating activated host DCs loaded with efficiently captured DCP-001 material. This proposed mode of action is illustrated in Figure 8.

DCP-001 expresses characterized and uncharacterized endogenous TAAs in combination with HLA class I/II molecules and co-stimulatory molecules of mature DCs. After intradermal (i.d.) injection, DCP-001, due to its allogeneic nature, stimulates local alloreactive T-cells. These T-cells can be activated by DCP-001 directly (direct pathway of allorecognition) or they might be activated indirectly by APCs that have taken up DCP-001 and present DCP-001-derived allo-MHC peptides to these T-cells (indirect pathway of allorecognition). It is known that the skin is rich in APCs, consisting of resident macrophages, Langerhans cells and dermal DCs, particularly cDC2 [39]. These DCP-001-capturing APCs might also already activate local T-cells recognizing DCP-001-derived TAAs. The activated alloreactive T-cells might support activation of these T-cells via a process known as “bystander T-cell activation”, wherein both types of T-cells interact with the same APCs [40], and via (non-cognate) “bystander T-cell help” through released cytokines [30,31] and/or enhanced release of DC-derived exosomes [41]. In addition, DCP-001 itself and uptake of DCP-001 by host APCs leads to activation/maturation of these cells, as was modelled by the effect of DCP-001 on imoDCs and migrating skin APCs. Co-culture with PBMCs or imoDCs caused the release of several pro-inflammatory cytokines and chemokines, e.g., RANTES, CXCL10 and CXCL16, known to attract and activate T-cells and NK cells [42,43,44], and GM-CSF, known to attract and activate myeloid cells (monocytes and DCs) [45] and to prevent the shift from mature DCs to immature CD14^+^ macrophage-like cells [17]. Recruited and activated host DCs, sometimes referred to as “bystander DCs”, have also been suggested to play an important role in the allogeneic cell-based intratumoral immune primer ilixadencel [3]. DCs that have captured DCP-001 can subsequently migrate to the draining LN. Importantly, for activation of TAA-reactive CD8^+^ CTLs, known to be crucial in the actual killing of tumor cells, cross-presentation of exogenously acquired antigen on MHC class I molecules by host APCs is required. Dendritic cells possess this cross-presenting activity, but one type of DC, the myeloid/conventional DC type 1 (cDC1), seems superior in this [46,47], and these cells are found in relatively high quantities in LNs [48]. Therefore, in the LNs, the migrated host DCs themselves can reactivate or prime lymphoid T-cells or they can transfer DCP-001 antigens to these cross-presenting cDC1 cells. This type of antigen transfer between LN DCs has been reported for other antigens, including tumor antigens [35,49,50,51].

In conclusion, the expression of many endogenous shared TAAs and the capacity of DCP-001 to induce a strong pro-inflammatory immune response accompanied by cell-bound antigen transfer to host DCs forms the rationale to investigate its use in different cancer types beyond AML and in combination with other immune therapies that require additional (TAA-specific) boosting of the immune system, such as immune checkpoint inhibitors or CAR T-cells.

## Data Availability

The data presented in this study are available on request from the corresponding author. The data are not publicly available due to proprietary confidential information.

## Supplementary Materials

The following are available online at https://www.mdpi.com/article/10.3390/cells10113233/s1, Table S1: List of antibodies and multiplex kits used throughout the study. Video S1: Dynamic interaction between DCP-001 and imoDCs.

## References

[1] de Gruijl T.D., van den Eertwegh A.J., Pinedo H.M., Scheper R.J. (2008). Whole-cell cancer vaccination: From autologous to allogeneic tumor- and dendritic cell-based vaccines. Cancer Immunol. Immunother..

[2] Yasuda T., Kamigaki T., Kawasaki K., Nakamura T., Yamamoto M., Kanemitsu K., Takase S., Kuroda D., Kim Y., Ajiki T. (2007). Superior anti-tumor protection and therapeutic efficacy of vaccination with allogeneic and semiallogeneic dendritic cell/tumor cell fusion hybrids for murine colon adenocarcinoma. Cancer Immunol. Immunother..

[3] Karlsson-Parra A., Kovacka J., Heimann E., Jorvid M., Zeilemaker S., Longhurst S., Suenaert P. (2018). Ilixadencel—An Allogeneic Cell-Based Anticancer Immune Primer for Intratumoral Administration. Pharm. Res..

[4] Anguille S., Van Tendeloo V.F., Berneman Z.N. (2012). Leukemia-associated antigens and their relevance to the immunotherapy of acute myeloid leukemia. Leukemia.

[5] Leaf R.K., Stroopinsky D., Pyzer A.R., Kruisbeek A.M., van Wetering S., Washington A., Ephraim A., Cole L., Morin A., Jain S. (2017). DCOne as an Allogeneic Cell-based Vaccine for Multiple Myeloma. J. Immunother..

[6] Vermeij R., Daemen T., de Bock G.H., de Graeff P., Leffers N., Lambeck A., ten Hoor K.A., Hollema H., van der Zee A.G., Nijman H.W. (2010). Potential target antigens for a universal vaccine in epithelial ovarian cancer. Clin. Dev. Immunol..

[7] van de Loosdrecht A.A., van Wetering S., Santegoets S., Singh S.K., Eeltink C.M., den Hartog Y., Koppes M., Kaspers J., Ossenkoppele G.J., Kruisbeek A.M. (2018). A novel allogeneic off-the-shelf dendritic cell vaccine for post-remission treatment of elderly patients with acute myeloid leukemia. Cancer Immunol. Immunother..

[8] Janssen L.L.G., Westers T.M., Rovers J., Valk P., Cloos J., de Gruijl T.D., Van de Loosdrecht A.A. (2019). Durable Responses and Survival in High Risk AML and MDS Patients Treated with an Allogeneic Leukemia-Derived Dendritic Cell Vaccine. Blood.

[9] Verdijk P., Aarntzen E.H., Lesterhuis W.J., Boullart A.C., Kok E., van Rossum M.M., Strijk S., Eijckeler F., Bonenkamp J.J., Jacobs J.F. (2009). Limited amounts of dendritic cells migrate into the T-cell area of lymph nodes but have high immune activating potential in melanoma patients. Clin. Cancer Res..

[10] Yewdall A.W., Drutman S.B., Jinwala F., Bahjat K.S., Bhardwaj N. (2010). CD8^+^ T cell priming by dendritic cell vaccines requires antigen transfer to endogenous antigen presenting cells. PLoS ONE.

[11] Stuart L.M., Ezekowitz R.A. (2005). Phagocytosis: Elegant complexity. Immunity.

[12] Liu Z., Roche P.A. (2015). Macropinocytosis in phagocytes: Regulation of MHC class-II-restricted antigen presentation in dendritic cells. Front. Physiol..

[13] Frydrychowicz M., Kolecka-Bednarczyk A., Madejczyk M., Yasar S., Dworacki G. (2015). Exosomes-structure, biogenesis and biological role in non-small-cell lung cancer. Scand. J. Immunol..

[14] Théry C., Duban L., Segura E., Véron P., Lantz O., Amigorena S. (2002). Indirect activation of naïve CD4^+^ T cells by dendritic cell-derived exosomes. Nat. Immunol..

[15] Sarkar S., Sabhachandani P., Stroopinsky D., Palmer K., Cohen N., Rosenblatt J., Avigan D., Konry T. (2016). Dynamic analysis of immune and cancer cell interactions at single cell level in microfluidic droplets. Biomicrofluidics.

[16] Ruben J.M., Bontkes H.J., Westers T.M., Hooijberg E., Ossenkoppele G.J., van de Loosdrecht A.A., de Gruijl T.D. (2014). In situ loading of skin dendritic cells with apoptotic bleb-derived antigens for the induction of tumor-directed immunity. Oncoimmunology.

[17] de Gruijl T.D., Sombroek C.C., Lougheed S.M., Oosterhoff D., Buter J., van den Eertwegh A.J., Scheper R.J., Pinedo H.M. (2006). A postmigrational switch among skin-derived dendritic cells to a macrophage-like phenotype is predetermined by the intracutaneous cytokine balance. J. Immunol..

[18] Garg A.D., Romano E., Rufo N., Agostinis P. (2016). Immunogenic versus tolerogenic phagocytosis during anticancer therapy: Mechanisms and clinical translation. Cell Death Differ..

[19] Hourigan C.S., Levitsky H.I. (2011). Evaluation of current cancer immunotherapy: Hemato-oncology. Cancer J. (Sudbury Mass.).

[20] Kloudova K., Hromadkova H., Partlova S., Brtnicky T., Rob L., Bartunkova J., Hensler M., Halaska M.J., Spisek R., Fialova A. (2016). Expression of tumor antigens on primary ovarian cancer cells compared to established ovarian cancer cell lines. Oncotarget.

[21] Brode S., Macary P.A. (2004). Cross-presentation: Dendritic cells and macrophages bite off more than they can chew!. Immunology.

[22] Nagata S., Tanaka M. (2017). Programmed cell death and the immune system. Nat. Rev. Immunol..

[23] Harms-Ringdahl M., Nicotera P., Radford I.R. (1996). Radiation induced apoptosis. Mutat. Res. Rev. Genet. Toxicol..

[24] Sanchez-Sanchez N., Riol-Blanco L., de la Rosa G., Puig-Kroger A., Garcia-Bordas J., Martin D., Longo N., Cuadrado A., Cabanas C., Corbi A.L. (2004). Chemokine receptor CCR7 induces intracellular signaling that inhibits apoptosis of mature dendritic cells. Blood.

[25] Fischer K., Voelkl S., Berger J., Andreesen R., Pomorski T., Mackensen A. (2006). Antigen recognition induces phosphatidylserine exposure on the cell surface of human CD8^+^ T cells. Blood.

[26] Sauter B., Albert M.L., Francisco L., Larsson M., Somersan S., Bhardwaj N. (2000). Consequences of cell death: Exposure to necrotic tumor cells, but not primary tissue cells or apoptotic cells, induces the maturation of immunostimulatory dendritic cells. J. Exp. Med..

[27] Birge R.B., Boeltz S., Kumar S., Carlson J., Wanderley J., Calianese D., Barcinski M., Brekken R.A., Huang X., Hutchins J.T. (2016). Phosphatidylserine is a global immunosuppressive signal in efferocytosis, infectious disease, and cancer. Cell Death Differ..

[28] Oh S.A., Wu D.-C., Cheung J., Navarro A., Xiong H., Cubas R., Totpal K., Chiu H., Wu Y., Comps-Agrar L. (2020). PD-L1 expression by dendritic cells is a key regulator of T-cell immunity in cancer. Nat. Cancer.

[29] D’Orsogna L.J., Roelen D.L., Doxiadis I.I.N., Claas F.H. (2010). Alloreactivity from human viral specific memory T-cells. Transpl. Immunol..

[30] Schenkel J.M., Fraser K.A., Beura L.K., Pauken K.E., Vezys V., Masopust D. (2014). T cell memory. Resident memory CD8 T cells trigger protective innate and adaptive immune responses. Science.

[31] Ariotti S., Hogenbirk M.A., Dijkgraaf F.E., Visser L.L., Hoekstra M.E., Song J.Y., Jacobs H., Haanen J.B., Schumacher T.N. (2014). T cell memory. Skin-resident memory CD8⁺ T cells trigger a state of tissue-wide pathogen alert. Science.

[32] Menares E., Galvez-Cancino F., Caceres-Morgado P., Ghorani E., Lopez E., Diaz X., Saavedra-Almarza J., Figueroa D.A., Roa E., Quezada S.A. (2019). Tissue-resident memory CD8(^+^) T cells amplify anti-tumor immunity by triggering antigen spreading through dendritic cells. Nat. Commun..

[33] Naeini M.B., Bianconi V., Pirro M., Sahebkar A. (2020). The role of phosphatidylserine recognition receptors in multiple biological functions. Cell Mol. Biol. Lett..

[34] Segawa K., Nagata S. (2015). An Apoptotic ‘Eat Me’ Signal: Phosphatidylserine Exposure. Trends Cell Biol..

[35] Ruhland M.K., Roberts E.W., Cai E., Mujal A.M., Marchuk K., Beppler C., Nam D., Serwas N.K., Binnewies M., Krummel M.F. (2020). Visualizing Synaptic Transfer of Tumor Antigens among Dendritic Cells. Cancer Cell.

[36] Caronni N., Piperno G.M., Simoncello F., Romano O., Vodret S., Yanagihashi Y., Dress R., Dutertre C.A., Bugatti M., Bourdeley P. (2021). TIM4 expression by dendritic cells mediates uptake of tumor-associated antigens and anti-tumor responses. Nat. Commun..

[37] Hayat S.M.G., Bianconi V., Pirro M., Jaafari M.R., Hatamipour M., Sahebkar A. (2020). CD47: Role in the immune system and application to cancer therapy. Cell. Oncol..

[38] Lian S., Xie R., Ye Y., Xie X., Li S., Lu Y., Li B., Cheng Y., Katanaev V.L., Jia L. (2019). Simultaneous blocking of CD47 and PD-L1 increases innate and adaptive cancer immune responses and cytokine release. EBioMedicine.

[39] Kashem S.W., Haniffa M., Kaplan D.H. (2017). Antigen-Presenting Cells in the Skin. Annu. Rev. Immunol..

[40] Sporri R., Reis e Sousa C. (2003). Newly activated T cells promote maturation of bystander dendritic cells but not IL-12 production. J. Immunol..

[41] Lindenbergh M.F.S., Koerhuis D.G.J., Borg E.G.F., van’t Veld E.M., Driedonks T.A.P., Wubbolts R., Stoorvogel W., Boes M. (2019). Bystander T-Cells Support Clonal T-Cell Activation by Controlling the Release of Dendritic Cell-Derived Immune-Stimulatory Extracellular Vesicles. Front. Immunol..

[42] Lapteva N., Huang X.F. (2010). CCL5 as an adjuvant for cancer immunotherapy. Expert Opin. Biol. Ther..

[43] Lebre M.C., Burwell T., Vieira P.L., Lora J., Coyle A.J., Kapsenberg M.L., Clausen B.E., De Jong E.C. (2005). Differential expression of inflammatory chemokines by Th1- and Th2-cell promoting dendritic cells: A role for different mature dendritic cell populations in attracting appropriate effector cells to peripheral sites of inflammation. Immunol. Cell Biol..

[44] Griffith J.W., Sokol C.L., Luster A.D. (2014). Chemokines and chemokine receptors: Positioning cells for host defense and immunity. Annu. Rev. Immunol..

[45] Becher B., Tugues S., Greter M. (2016). GM-CSF: From Growth Factor to Central Mediator of Tissue Inflammation. Immunity.

[46] Collin M., Bigley V. (2018). Human dendritic cell subsets: An update. Immunology.

[47] Haniffa M., Shin A., Bigley V., McGovern N., Teo P., See P., Wasan P.S., Wang X.N., Malinarich F., Malleret B. (2012). Human tissues contain CD141^hi^ cross-presenting dendritic cells with functional homology to mouse CD103^+^ nonlymphoid dendritic cells. Immunity.

[48] Granot T., Senda T., Carpenter D.J., Matsuoka N., Weiner J., Gordon C.L., Miron M., Kumar B.V., Griesemer A., Ho S.H. (2017). Dendritic Cells Display Subset and Tissue-Specific Maturation Dynamics over Human Life. Immunity.

[49] Allan R.S., Waithman J., Bedoui S., Jones C.M., Villadangos J.A., Zhan Y., Lew A.M., Shortman K., Heath W.R., Carbone F.R. (2006). Migratory dendritic cells transfer antigen to a lymph node-resident dendritic cell population for efficient CTL priming. Immunity.

[50] Gurevich I., Feferman T., Milo I., Tal O., Golani O., Drexler I., Shakhar G. (2017). Active dissemination of cellular antigens by DCs facilitates CD8(^+^) T-cell priming in lymph nodes. Eur. J. Immunol..

[51] Borst J., Ahrends T., Bąbała N., Melief C.J.M., Kastenmüller W. (2018). CD4(^+^) T cell help in cancer immunology and immunotherapy. Nat. Rev. Immunol..

