# Supplementary material for: Transfer of Cellular Content from the Allogeneic Cell-Based Cancer Vaccine DCP-001 to Host Dendritic Cells Hinges on Phosphatidylserine and Is Enhanced by CD47 Blockade
Source: Cells. 2021 Nov 19;10(11):3233. doi: 10.3390/cells10113233 (PMC8625408; doi:10.3390/cells10113233)
Supplement: Supplementary file 1 [file cells-10-03233-s001.zip › cells-1439604-suppl/Table S1.pdf]

Table S1. List of antibodies and multiplex kits used throughout the study

Antibodies	Source	Catalog No.
**Used as single antibodies:**		
Anti-human CD1a FITC (HI149)	BD Biosciences	555806
Anti-human CD1a APC (HI149)	BioLegend	300110
Anti-human CD3 APC (UCHT1)	BD Biosciences	555335
Anti-human CD11c APC (B-ly6)	BD Biosciences	559877
Anti-human CD14 APC-H7 (MϕP9)	BD Biosciences	560180
Anti-human CD14 FITC (MϕP9)	BD Biosciences	345784
Anti-human CD14 APC-fire (63D3)	BioLegend	367120
Anti-human CD19 APC (HIB19)	BD Biosciences	555415
Anti-human CD34 PE (581)	BD Biosciences	555822
Anti-human CD34 PerCP-Cy5.5 (581)	BioLegend	343522
Anti-human CD40 FITC (5C3)	BD Biosciences	555588
Anti-human CD70 PE (113-16)	BioLegend	355104
Anti-human CD80 FITC (L307.4)	BD Biosciences	557226
Anti-human CD80 APC (2D10)	BioLegend	305220
Anti human CD80 BV510 (2D10)	BioLegend	305234
Anti-human CD83 PE (HB15a)	Beckman Coulter	PNIM2218U
Anti-human CD83 APC (HB15e)	BD Biosciences	551073
Anti-human CD83 BV421 (HB15e)	BioLegend	305324
Anti-human CD86 PE (FUN-1)	BD Biosciences	555658
Anti-human CD86 APC (FUN-1)	BD Biosciences	555660
Anti human CD86 PE-Cy7 (BU63)	BioLegend	374210
Anti-human CD209 APC (DCN46)	BD Biosciences	551545
Anti-human CD274 APC (MIH1)	BD Biosciences	563741
Anti-human CD304 APC	BioLegend	354506
Anti-human HLA-ABC APC (W6/32)	BioLegend	311410
Anti-human HLA-DR PerCP-Cy5.5 (G46-6)	BD Biosciences	552764
Anti-human HLA-DP/DQ/DR FITC (Bu26)	GeneTex	GTX43442
Anti-human HLA-DP/DQ/DR APC (Tü39)	BioLegend	361714

Mouse IgG1 PE, Isotype Control (X40)	BD Biosciences	345816
Mouse IgG1 FITC, Isotype Control (X40)	BD Biosciences	345815
Mouse IgG1 APC, Isotype Control (MOPC-21)	BD Biosciences	554681
Mouse IgG2a APC, Isotype Control (MOPC-173)	BioLegend	400220
Mouse IgG2b FITC, Isotype Control (MPC-11)	BD Biosciences	559532
Mouse IgG2b APC, Isotype Control (MPC-11)	BioLegend	400320
Purified Mouse Anti-human CD36 (CB38)	BD Biosciences	555453
Purified Mouse Anti-human CD47 (CC2C6)	BioLegend	323102
Purified Mouse Anti-human CD204 (7C9C20)	BioLegend	371902
Purified Mouse Anti-human LOX-1 (23C11)	Hycult Biotech	HM2138
Purified Rabbit Anti-human calreticulin	Abcam	ab2907
Purified Mouse Anti-human CD91 (A2MR-α2)	BD Biosciences	550495
Purified Mouse Anti-human CD102 (ICAM-2)	BioLegend	328502
Purified anti-human CD172a/b (SIRPα/β) (SE5A5)	BioLegend	323802
Purified Mouse Anti-human CD209 (DCN46)	BD Biosciences	551186
Purified Mouse Anti-human CD282 (11G7)	BD Biosciences	558317
Purified Mouse Anti-human CD284 (HTA125)	BioLegend	312802
Mouse IgG1, Isotype Control (P3.6.2.8.1) (including Azide)	Thermo Fisher Scientific	16-4714-82
**Targets tested by customized multiplex kits:**		
**3-plex**: CCL5/Rantes; CXCL10, IL-8<br><br>**8-plex**: IL-1β, CCL2; CXCL9; GM-CSF; IFN-γ; IL-2; IL-6; TNF-α	eBioscience	EPX030-00000-801<br><br>EPX080-00000-801
**19-plex:** CCL2; CCL3; CCL4; CX3CL1; CXCL1; CXCL13; CXCL16; CXCL2; CXCL9; GM-CSF; IFN-γ; IL-10; IL-12/IL23p40; IL-18; IL-1β; IL-2; IL-5; IL-6; TNF-α<br><br>**3-plex**: CCL5/Rantes; CXCL10, IL-8<br><br>**3-plex**: TGFβ1; TGFβ2; TGFβ3<br><br>**1-plex**: IL12p70	Bio-Techne	LXSAHM-19<br><br>LXSAHM-3<br><br>LXSAHM-3(TGFb)<br><br>LXSAHM-1
